# Efficacy of Herbal Drugs in Human Diseases and Disorders

**DOI:** 10.1155/2014/273676

**Published:** 2014-06-18

**Authors:** Suaib Luqman, Syed Ibrahim Rizvi, André-Michael Beer, Sunil Kumar Khare, Pinar Atukeren

**Affiliations:** ^1^Academy of Scientific and Innovative Research (AcSIR), Molecular Bioprospection Department, CSIR-Central Institute of Medicinal and Aromatic Plants, Lucknow, Uttar Pradesh 226015, India; ^2^Department of Biochemistry, Faculty of Science, University of Allahabad, Allahabad, Uttar Pradesh 211002, India; ^3^True Naturopathy Department, Blankenstein Clinic, Ruhr University, Im Vogelsang 5-11, D-45527 Hattingen, Bochum, Germany; ^4^Enzyme and Microbial Biochemistry Laboratory, Department of Chemistry, Indian Institute of Technology Delhi (IITD), New Delhi 110016, India; ^5^Department of Medical Biochemistry, Cerrahpasa Medical Faculty, Istanbul University, Istanbul 34303, Turkey

According to a ballpark figure by World Health Organization, more than three-quarters of population of the developing nations exploit herbal medicine for their primary health care. Herbal drugs and their constituents have advantageous effects on long-term fitness and can be used to efficiently treat human diseases or disorders. In veracity, herbs frequently include various active ingredients that imaginably have drug allying commotion in the body. Bringing into play, herbs are often more affordable than procuring expensive contemporary pharmaceuticals to take care of ailments. A large number of herbs have proved their usefulness in managing various illnesses. Latest advances in biology and medicine have introduced new technologies to study the biological significance of herbal drugs in various human diseases and disorders. Hence it is important to understand the mechanism(s) of herbal drug action for the knowledge and development of successful therapies.

The current special issue brings together several exciting papers defining the efficacy of herbal drugs in an assortment of human diseases and disorders.

J. Li et al. have tested the effects and mechanism of Bufei Yishen formula (BYF) in a rat chronic obstructive pulmonary disease (COPD) model and provided the basis for mechanisms of BYF on COPD and new therapeutic drug targets.

X. Yu et al. have reported the treatment of Si-Jun-Zi decoction, a famous Chinese medicine that promotes the restoration of intestinal function after obstruction by regulating intestinal homeostasis.

Tong xinluo (TXL), a Chinese herbal compound, has been used in China with traditional therapeutic efficacy in patients with diabetic nephropathy (DN). N. Zhang et al. demonstrated that TXL successfully inhibits TGF-*β*1-induced epithelial-to-mesenchymal transition in DN, which may account for the therapeutic efficacy in TXL-mediated renal protection.

W. Zhang et al. explored the effects of Jian-Pi-Zhi-Dong decoction (JPZDD) on Tourette syndrome (TS) by investigating the expression of gamma-aminobutyric acid (GABA) and its type A receptor (GABAAR) in the striatum of a TS mice model induced by 3,3′-iminodipropionitrile (IDPN) treatment. It was revealed that JPZDD alleviates TS symptoms which may be associated with GABAAR expression downregulation in striatum thereby controlling GABA metabolism.

TZQ-F has been traditionally used as a traditional Chinese medicine formula for the treatment of diabetes. H. Yuhong et al. compared the pharmacologic effects and gastrointestinal adverse events between TZQ-F and acarbose in Chinese healthy volunteers.

T. Numata et al. reported the treatment of posttraumatic stress disorder (PTSD) using the traditional Japanese herbal medicine Saikokeishikankyoto in survivors of the great east Japan earthquake and tsunami. The traditional medicine may be a valid choice for the treatment of psychological and physical symptoms in PTSD patients.

Y. Ewnetu et al. evaluated the synergetic antimicrobial effect of Ethiopian honey and ginger powder extract mixtures on standard and resistant clinical isolates of* Staphylococcus aureus*,* Escherichia coli,* and* Klebsiella pneumoniae*. The combination has the potential to serve as cheap source of antibacterial agents especially for the drug resistant strains.

Herbs are now gaining much attention as the main source of effective drugs for lowering serum lipids and lipid peroxidation. M. Rafieian-kopaei et al. assessed the impact of* Ferulago angulata* on serum lipid profiles and on levels of lipid peroxidation in male Wistar rats. Administration of a hydroalcoholic extract reduces the serum levels of total cholesterol, triglycerides, and LDL comparable to atorvastatin (a standard lipid lowering drug).

A-J. Lee et al. investigated the absolute counts and percentages of peripheral blood (PB) lymphocyte subtypes in end-stage cancer patients before and after localized radiotherapy (RT) and after oral administration of Bojungikki-tang water extract (BJITE) and to evaluate the changes mediated by RT and BJITE. It was observed that immune deterioration occurs after RT and administration of BJITE was found effective in its restoration.

T. Shimizu provides a good overview of efficacy of Kampo medicine (KM) in treating atopic dermatitis (AD). KM is a traditional herbal medicine in Japan and has a long history and plays an important role in the prevention and treatment of various diseases including AD.

Y. Ma et al. explored the efficacy of herb-partitioned moxibustion in patients with diarrhoea-predominant irritable bowel syndrome (DPIBS) and suggest it as a promising, efficacious, and well-tolerated treatment for patients.

Melinjo (*Gnetum gnemon* L.) seed extract containing transresveratrol (3,5,4′-trihydroxy-trans-stilbene) and other derivatives exerts diverse beneficial effects. H. Konno et al. reported melinjo seed extract decreases serum uric acid levels in nonobese Japanese males regardless of insulin resistance and may improve lipid metabolism by increasing HDL cholesterol.

We anticipate that readers and scientific fraternity working in the area of* herbal drugs* will find not only the updated review on the subject but also a compilation of precise data on the efficacy of herbal drugs in some lifestyle diseases and disorders with their suggested mechanism of action.

*Suaib Luqman* *Suaib Luqman* *Syed Ibrahim Rizvi* *Syed Ibrahim Rizvi* *André-Michael Beer* *André-Michael Beer* *Sunil Kumar Khare* *Sunil Kumar Khare* *Pinar Atukeren* *Pinar Atukeren*

